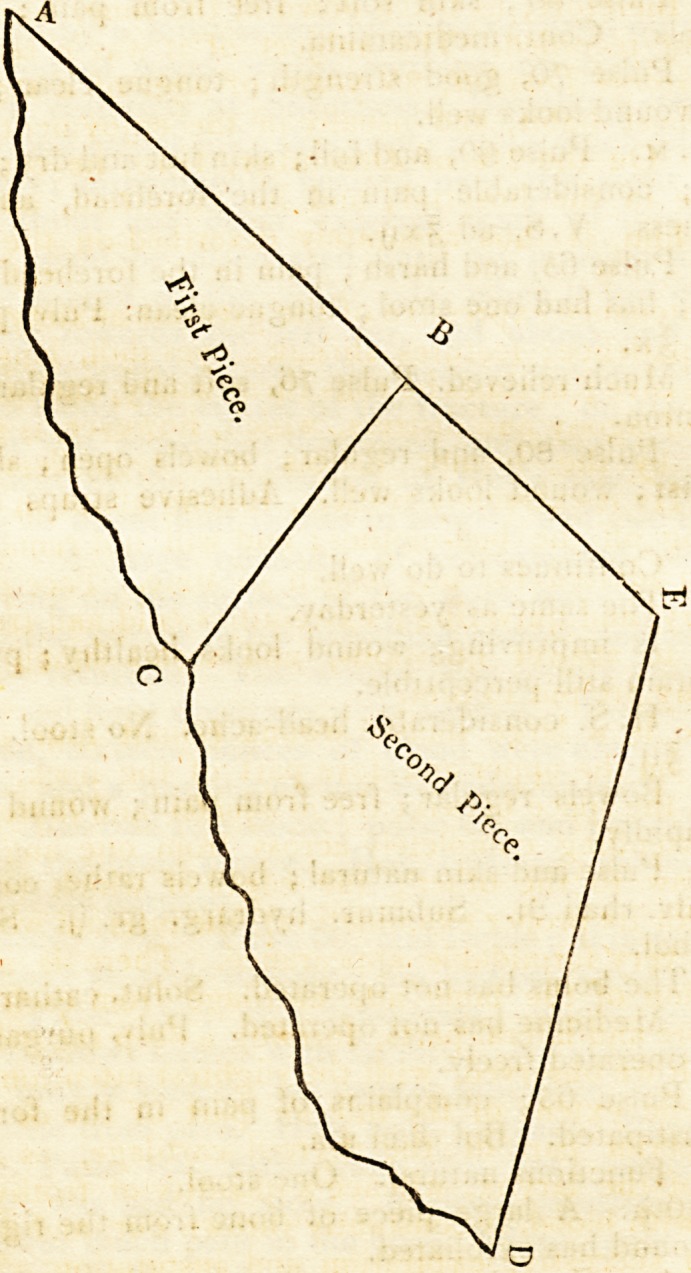# Dr. Dobson's Case of Fracture of the Cranium

**Published:** 1817-11

**Authors:** D. Quarrier


THE
### Medico-Chirurgical
### Journal and Review.

VOL. IV.] NOVEMBER, 1817. [NO. 23.

### PART I.

### ORIGINAL COMMUNICATIONS.

——— quid utile ———

*To the Editors of the Medico-Chirurgical Journal & Review.*

GENTLEMEN,

*Fort Monckton, Aug. 14th,* 1817.

HEREWITH I enclose a Case of Fracture of the Cranium, transmitted to me by Dr. Dobson, Surgeon, Royal Marines, Chatham, which may be useful to some of your readers, as it fully exhibits one of those instances which so frequently occur in the practice of surgery, where nothing but a degree of boldness, the result of chirurgical experience, and scientific knowledge of the injured parts, can possibly save the life of the patient.

The man was carried on board the Argonaut, during the absence of Dr. Douglass*, the able surgeon of that ship; when Dr. Dobson, who was officiating for him, proceeded in the manner described; and from the quantity of coagulum removed from pressing on the brain, and the general derangement of the vital functions, we cannot hesitate in believing, that this man was rescued from the brink of the grave by the decisive measures pursued. The case is merely a transcript of the man's ticket, and Dr. Douglass, on whom the subsequent treatment devolved, deserves the highest praise for his unremitted attention to him during the curative process. The accompanying Sketch is exactly the size and shape of the portions of bone removed;

* Dr. Douglass was called to town at the time the accident occurred, to receive from the hands of the royal patron of the Humane Society, a medal which had been awarded to him by that society, for having saved by bronchotomy, the life of a fellow creature.

and the man, I am happy to say, is now well, and discharged from the Hospital Ship.

I am, Gentlemen,

Very faithfully yours,

D. QUARRIER, M. D.

April 3d, 1817. Joseph Brown, Seaman, ætat. 25, was received on board His Majesty's Ship Argonaut, at Chatham, this afternoon, in a state of total insensibility, occasioned by a most severe blow he received on the head from a log of wood, whilst working on board a naval transport yesterday at Sheerness. Breathing stertorous; pulse 60, and rather full; skin cool; pupils much dilated and motionless; integuments bruised over the right parietal bone, without any other external mark of injury. No fracture could be discovered until an incision was made from the wound of the right side, when it was exhibited extending towards the left ear, and about half an inch behind the coronal suture; the incision was carried across the head along the course of the fracture; on the left side the bone was somewhat depressed, and two pieces were taken away by the application of Hey's saw, leaving an opening of about four inches in length, and two inches in breadth. A large quantity of extravasated blood was found lying on the surface of the dura mater; and on its removal the stertor in his breathing ceased, and sensibility gradually returned. The wound was gently brought together, and light dressings were applied. Previous to the operation, sixteen ounces of blood were abstracted from the arm, and a purging clyster was administered.

4th. Has had two stools; pulse 66, and irregular; pulsation of the brain strong. Hab. hydrarg. submur. gr. x. Rept. enema purgans.

6 o'clock, P. M. Pulse 60, and feeble; skin temperate; pupils more sensible to light: has had one stool.

5th. Pulse 110; skin temperate; pupils sensible to light; swallows liquids with facility. No stool during the night; tongue loaded; he cannot articulate, but seems to suffer little from pain. Hab. pulv. purgant. Rept. enema.

H. S. Pulse 100, rather full; skin hot; tongue cleaner and dry; has had no stool. Answers questions at times; says he has much pain in the head. Rept. pulv. purgans et enema. V. S. ad ℥ xij.

10, P. M. Has had two copious stools; pulse 100, and feeble; extremities cool. Answers questions distinctly; says he has little pain; seems restless and disturbed.

℞. Tinct. opii Spt. æther nitros āā ♏ xl. Aq. font. ℥ij M ft. haust stat sumendus.

6th. 10, A. M. Pulse 80, moderate strength; skin temperate; face a little flushed; two stools; sensible; says his vision is a little impaired; wound somewhat inflamed about the edges, and painful to the touch. Appl. cataplasm emolliens.

H. S. Pulse 80, rather full; skin hot; tongue dry; much thirst; wound almost free from pain; sleeps sound at intervals.

7th. Pulse 80; skin soft; free from pain; has had two stools. Cont. medicamina.

8th. Pulse 70, good strength; tongue clean; bowels open; wound looks well.

10, P. M. Pulse 90, and full; skin hot and dry; tongue whitish; considerable pain in the forehead, and great restlessness. V. S. ad ℥xij.

9th. Pulse 65, and harsh; pain in the forehead; much relieved; has had one stool; tongue clean. Pulv. purgans. V. S. ad ℥x.

H. S. Much relieved. Pulse 76, soft and regular. Cont. medicamina.

10th. Pulse 80, and regular; bowels open; skin soft and moist; wound looks well. Adhesive straps are applied.

11th. Continues to do well.

12th. The same as yesterday.

13th. Is improving; wound looks healthy; pulsation of the brain still perceptible.

14th. H. S. considerable head-ache. No stool. Solut. cathart. ℥ij.

16th. Bowels regular; free from pain; wound granulating rapidly.

18th. Pulse and skin natural; bowels rather costive.

℞. Pulv. rhæi ℈i. Submur. hydrarg. gr. ij. Syrup q. s. ut ft. bol.

H. S. The bolus has not operated. Solut. cathart. ℥ij.

19th. Medicine has not operated. Pulv. purgans.

H. S. operated freely.

23d. Pulse 65; complains of pain in the forehead; belly constipated. Bol rhæi u.a.

24th. Functions natural. One stool.

May 10th. A large piece of bone from the right side of the wound has exfoliated.

June 7th. Considerable pain of the forehead with vertigo; pulse 76; bowels regular. V. S. ad ℥xvj.

H. S. The pain continues unrelieved; pulse 90, full and strong; has had no stool since the morning. Rept. V. S. ad ℥xij. Solut. cathart. ℥ij.

8th. Pain less severe; bowels free; wound looks healthy and clean; pulse 80, and full. Rept. V. S. ad ℥xij.

9th. Pain of the head quite relieved.

July 11th. Right side of the wound cicatrized; at times small spicula of bone come to the surface. On the left side there is still about the breadth of the finger to skin over; undulation of the brain evident; functions natural.

That portion of bone included in the figure A B C was on the left, and depressed; and was removed by making a cut with Hey's saw from A to B, and another from B to C, each ending in the fracture; after which, finding compression upon the brain from coagulum lodged under the portion of bone B E D C, the cut A B was carried on to E, when it was met by another cut from the fracture at D.

## Figures and Tables

**Figure f1:**